# Analysis of the Economic-Financial Efficiency of Gout Treatment in Elderly Patients with Comorbidities in the Republic of Moldova

**DOI:** 10.31138/mjr.20230725.ao

**Published:** 2023-07-25

**Authors:** Larisa Rotaru, Liliana Groppa, Eugeniu Russu, Victor Cazac, Svetlana Agachi, Codreanu Cătălin, Larisa Spinei, Oleg Arnaut, Oxana Sârbu, Cornelia Cornea

**Affiliations:** 1 Department of Internal Medicine, Rheumatology and Nephrology, Universitatea de Stat de Medicina si Farmacie Nicolae Testemitanu, Chişinău, Moldova,; 2 Rheumatology Laboratory, Timofei Mosneaga Republican Clinical Hospital, Chişinău, Chişinău, Moldova,; 3 Faculty of Dentistry, Department 5 - Internal Medicine (Cardiology, Gastroenterology, Hepatology, Rheumatology, Geriatrics), Family Medicine, Occupational Medicine, Faculty of Medicine, Carol Davila University of Medicine and Pharmacy, Bucuresti, Romania,; 4 Department of Social Medicine and Management “Nicolae Testemitanu”, Universitatea de Stat de Medicina si Farmacie Nicolae Testemitanu, Chişinău, Moldova,; 5 Information Laboratory, Universitatea de Stat de Medicina si Farmacie Nicolae Testemitanu, Chişinău, Moldova

**Keywords:** gout, statistics, comorbidity

## Abstract

**Introduction:**

The costs of treating an elderly patient with gout are largely related to the treatment of concomitant pathological conditions and complications. Determining the costs of treating the disease is made by clinical and economic analysis, the task of which is to calculate the “cost of the illness”.

**Material and methods:**

A descriptive, selective study of 237 patients with gout. In order to study the clinical characteristics of gout in the elderly, all those included in the study was divided according to age at the time of examination: group I made up of patients with gout up to and including 59 years n=91, average age 48.1±7.4 years (from 30 years to 59 years), group II – over the age of 60 years inclusive n=146, average age 69.2±6.0 years, (60–86 years, p<0.01).

**Results:**

For calculations, the hospitalisation rate during the year was used, which had significant differences in groups: in group I it was 0.7; in group II – 1.2 (p=0,001). The costs of clinical management of gout and each of the diseases that accompany it for 1 calendar year have been determined.

**Conclusions:**

The direct costs for the treatment of patients with gout, calculated taking into account National Clinical Protocol and comorbid pathology, increase significantly with the age of the patients: the average cost per year of the treatment of gout in patients of mature age was 1337 Euro without and 7320 Euro taking into account the concomitant pathology, and in the elderly 2067 Euro and, respectively, 15230 Euro.

## INTRODUCTION

In recent decades, there has been a growing interest in the economic comparative assessment of health technologies in the field of diseases,^1–3,17^ the impact of diseases on the health system budget, including gout.^3–5^

The economic burden of gout is difficult to overestimate: among the United States of America (USA) working population, people suffering from gout have an average of 5 days of disability per year than people who do not suffer from gout.^1,3,6^ The additional annual cost of caring for a patient with gout exceeds $3,000 compared to the cost of people who do not suffer from gout, which puts gout on a par with the most economically burdensome diseases such as migraine and Parkinson’s disease.^2,7–9^

The costs of treating an elderly patient with gout are largely related to the treatment of concomitant pathological conditions and complications.^5,7,10,18^ Determining the costs of treating the disease is made by clinical and economic analysis, the task of which is to calculate the “cost of illness” (COI). Cost analysis is the most important element of clinical and economic analysis, has an independent significance, including the definition of the following indicators.^1–3,7,8,11^

*Direct costs* are the costs incurred by the healthcare system, the patient or other payer, by society as a whole directly in the process of providing healthcare.^2–5^ Direct costs include medical and non-medical costs.

*Direct medical costs* are direct costs for the provision of healthcare and costs associated with the provision of medical care, including those that are not directly related to the patient (work of the hospital administration, costs of electricity and heating, etc.).^2,4,6^

*Direct non-medical costs* are the expenditure of other sectors of the economy (eg, social services) and personal expenses of the patient and his relatives not directly related to healthcare: expenditure related to the payment for arrival at the place of treatment, with lifestyle changes due to illness.^3^

*Indirect costs* are the costs of resources that were not created due to illness, the loss of society due to temporary and persistent disability and premature death.^5,6^

*Intangible costs* are losses of patients due to suffering caused by diseases that occur but are poorly valued in monetary terms.^4,12^

With the simultaneous implementation of medical services, the question arises of the impact of the number of these services on the value of costs. In the economy, the concept of marginal cost is used, which shows additional costs when the volume of production increases by one unit of utility.^3–5,15–16^

The determination of average and specific (marginal) costs (costs with an increase in the number of services provided) is an important mechanism that allows you to optimize the provision of healthcare and anticipate it for a long period of time.^6,8,9^ A necessary condition for determining the costs of simultaneous healthcare in the treatment of several diseases is to take into account the fact that fixed (mainly indirect) medical costs are relatively low, and the ratio of 1/3 becomes incorrect. When developing the Nomenclature of Services in the Field of Health, the authors empirically obtained the values of the correction coefficient of the cost indicators (margin coefficient) applied in the case of calculating the costs for a complex medical service consisting of simple services performed simultaneously.^1–4,7,11–14^ It is proposed to introduce this coefficient in the form of a multiplier to the sum of the costs for simple medical services that are part of a complex service.

## OBJECTIVE OF THE STUDY

Analysis of economic and financial efficiency in the treatment of gout in elderly patients with comorbidities.

## MATERIALS AND METHODS

A descriptive, selective study of 237 patients with gout (average age for the men 60±8.0 years and for the women 63±9.0 years) is conducted. The study was carried out in accordance with the requirements of the Ministry of Health for “Clinical and financial-economic research” within the postdoctoral scientific program at the Discipline of Rheumatology and Nephrology of the “Nicolae Testemiţanu” State University of Medicine and Pharmacy of the Republic of Moldova.

From the database of the Departments of Arthrology, Rheumatology, and Nephrology of the “Timofei Mosneaga” Republican Clinical Hospital were extracted the clinic-paraclinical data and the performed treatment of patients with gout, including 658 patients with gout observed in the period 2015 – 2022, of which 237 patients who meet the criteria of the study were selected. The diagnosis of gout in the database was carried out in accordance with the classification criteria for gout according to ACR and EULAR 2015.^3–5^ Determining the costs of treatment of a patient with gout in groups I and II. In order to study the clinical characteristics of gout in the elderly, all those included in the study were divided according to age at the time of examination: group I was made up of patients with gout up to and including 59 years (n=91, average age 48.1±7.4 years [from 30 to 59 years]), group II – over the age of 60 years inclusive (n=146 average age 69.2±6.0 years [60–86 years, p<0.01]). This age distribution is based on the recommendations of the European Consensus for patients with comorbidities. The current cost was accounted from data from a single Centre and, also, all costs are calculated from the data of compulsory medical insurance for all citizens of our country in a centralised form.

The raw data was processed in SPSS version 26.0.

## RESULTS

For the calculation we used tariffs for medical services provided under the National Clinical Protocols (NCP) which were derived from Recommendations of Guidelines of EULAR for patients with gout, taking into account comorbidities: arterial hypertension, coronary heart disease, chronic heart failure (CHF), nephrolithiasis, chronic kidney disease (CKD), diabetes mellitus type 2 (DM 2). Costs were determined for patients with gout per calendar year in the 2 study groups: in group I it was 0.7; in group II it was 1.2 (p = 0.001), including comorbid pathology (**Table 1**). The NCP for each pathology were used separately for calculation.

**Table 1.** Costs of treatment of gout and each concomitant disease for 1 calendar year.

**Disease**	**Costs of outpatient treatment, Euro (% of total costs)**		**Inpatient treatment costs, Euro (% of total costs)**		**Total costs, Euro (100%)**	
	**Group I**	**Group II**	**Group I**	**Group II**	**Group I**	**Group II**
**Gout**	314 (23)	314 (15)	1023 (77)	1754 (85)	1337	2068
**Arterial hypertension**	104 (14)	104 (9)	615 (86)	1055 (91)	720	1159
**IHD**	132 (3)	132 (2)	4940 (97)	8470 (98)	5073	8602
**CHF**	264 (15)	264 (9)	1498 (85)	2568 (91)	1762	2832
**Urolithiasis**	184 (3)	184 (2)	2102 (97)	3604 (98)	2163	3665
**CKD**	1288 (18)	1288 (12)	5698 (82)	9768 (88)	6986	11057
**DM 2**	2270 (18)	2270 (67)	653 (22)	1120 (33)	2923	3390

IHD: ischemic heart disease; CHF: chronic heart failure; CKD: chronic kidney disease; DM 2: diabetes mellitus type 2.

In group I, the cost of treatment was 13% for outpatient care and 87% for inpatient care. In group II, the cost of inpatient care was slightly higher than in group I (8% for outpatient care and 92% for inpatient care), which was associated with a higher frequency of hospitalization and comorbid conditions. Inpatient treatment for chronic heart failure and ischemic heart disease was the most expensive of the comorbidities.

The most expensive of the diseases considered was the inpatient treatment of CKD and coronary artery disease.It has been calculated that treatment of gout with comorbidities costs an average of 5.5 to 29.5% of total costs per year (**Table 2**).

**Table 2.** The cost of providing medical assistance for gout and concomitant diseases.

**Disease**	**General costs for a case of hospital treatment**	**% of total costs**		**Annual overall costs in group I, % of total costs**	**Overall costs for one year in Group II, % of total costs**
		**Group I**	**Group II**		
**Gout**	434	22.7	25.2	336 (25.1)	553 (26.7)
**Arterial hypertension**	279	27.2	28.9	202 (28.1)	342 (29.5)
**IHD**	577	7.9	8.05	410 (8)	699 (8.1)
**CHF**	576	22.9	24.4	421 (23.9)	710 (25)
**Urolithiasis**	319	10.3	10.45	277 (10.5)	386 (10.5)
**CKD**	543	5.4	5.9	387 (5.5)	659 (6)
**DM2**	229	5.5	8.1	236 (8.1)	351 (10.3)

IHD: ischemic heart disease; CHF: chronic heart failure; CKD: chronic kidney disease; DM 2: diabetes mellitus type 2.

In order to calculate the costs of treating a conditioned patient for 1 year, taking into account comorbid pathology, it was proposed to use a decreasing marginality coefficient. The coefficient is of a nonlinear nature, its value was empirically derived and entered the Nomenclature of Works and Services in the Field of Health in 2017.

The need to apply the marginality coefficient in the presence of comorbidity can be justified by comparing the different calculation methods: without and using this coefficient.

For example, consider the option of having 5 concomitant diseases in a patient with gout over the age of 60: arterial hypertension, coronary artery disease, chronic heart failure, urolithiasis, and diabetes mellitus type 2.

When summing up all the costs of treating 5 diseases, except for gout, the total costs projected per patient for 1 year are 21718 Euro. With the introduction of the marginality coefficient, the projected costs are only 13031 Euro.

## DISCUSSIONS

Gout is a disease with a high comorbid background, accounting for the costs of treating concomitant diseases is advisable and necessary. For example, in some currently available clinical and economic studies, the assessment of comorbid pathology in gout was more often carried out using self-determination, which can distort the results.^8–11,19^ In the practice of health care, when providing assistance to elderly patients, accounting for the costs of concomitant pathology is a prerequisite.

The costs of treating gout with concomitant pathology exceeded the costs of treating a patient with gout 8 times, which confirms the need to take them into account for the objectivity of clinical and economic research.^3,9,13,15,18^ However, many categories of elderly citizens receive medicines preferentially, in part or in full, at the expense of the budget, and the costs of outpatient treatment for diseases of the cardiovascular system, diabetes mellitus are quite high.^6,15,19^ The costs of inpatient treatment according to this method are made by multiplying the individual fare by the day-bed and the number of days depending on the NCP, that is, calculated for a case of inpatient treatment. As the number of cases of inpatient treatment over the same period of time varies considerably from patient to patient, the cost of inpatient treatment during the year requires determining the multiplicity of service provision.^7,11,17^ The cost analysis method is convenient for mutual settlements between a particular manufacturer and the recipient of medical services, since the actual costs per visit to the clinic or a day of inpatient in institutions have different values. But this method does not answer the question of the cost of a particular nosology, as well as the contribution of concomitant diseases to its formation.^2–4,20^

In the study, the number of services was determined according to NCP, so the cost values are quite impressive, but they include the entire amount of medical care provided, including expensive examinations, procedures, surgical interventions, the use of highly effective drugs. For example, the calculated costs for NCP in elderly patients with concomitant hypertension, nephrolithiasis and coronary artery disease are 12403 Euro.

A separate discussion requires on the use in the study of such a value as the average number of hospitalisations per year. It should be clarified that according to the literature,^6 ,9,11^ there is no indicator such as the number of cases of hospitalized treatment per year. All indicators are correlated with bed-days and bed rotation, hospitalization for each disease.

In general, the proposed costing method allows for its widespread use in cost forecasting, conducting clinical and economic analyses, and also serves as justification for updating NCP. The dependence of the value of direct costs per case of inpatient medical care for elderly patients with gout on different calculation methods presented in the paper, determines the need for their further improvement and optimization.

The most real global catastrophe that threatens our population is associated with an ageing population: very soon, the number of people requesting expenditure that exceeds their own contribution to the economy will represent a significant part of the citizens of the developed countries of the world. The main burden on the health system will be related to the treatment of non-communicable diseases dependent on age, which are rapidly accumulating in the elderly society. The gap between health needs and the level of its funding will increase. The main task today is to estimate the amount of costs for the treatment of elderly patients, predict them and choose the least expensive and most effective tactics for their management and to prevent the development of chronic non-communicable diseases. Perhaps the study of concomitant diseases in patients with gout will determine the most effective measures to prevent their development, ensure healthy longevity, extend the working age of the population, and reduce the economic costs of the state.

## CONCLUSIONS

1. The average cost of one year of gout treatment for group I patients was 133712 Euro excluding comorbidities and 7320 Euro with comorbidities, and for group II - 2067 Euro and 15230 Euro respectively.
2. The frequency of hospitalisations during the year in patients with gout elderly (1.2 cases per year) increases significantly compared to patients with gout of working age (0.7 cases per year), which significantly affects the cost of health care.
3. The costs per case treated in the hospital for elderly gout patients, calculated in different ways, which determines the opportunity of their improvement and subsequent optimisation.
